# Supplementary material for: Upregulation of miR145 and miR126 in EVs from Renal Cells Undergoing EMT and Urine of Diabetic Nephropathy Patients
Source: Int J Mol Sci. 2022 Oct 11;23(20):12098. doi: 10.3390/ijms232012098 (PMC9603196; doi:10.3390/ijms232012098)

Upregulation of miR145 and miR126 in EVs from renal cells undergoing EMT and urine of diabetic nephropathy patients.

Veronica Dimuccio[1], Linda Bellucci[2], Marianna Genta[1], Cristina Grange[3], Maria Felice Brizzi[3], Maddalena Gili[1], Sara Gallo[1], Maria Laura Centomo[1], Federica Collino[2,4 #] and Benedetta Bussolati[1#*].

SUPPLEMENTARY MATERIAL

Supplementary Table S1

Supplementary Table S2

Supplementary Figure S1

Supplementary Table S1. Oligonucleotide primers sequences for miRNA detection.

miR21	F- TAGCTTATCAGACTGATGTTGA
miR24	F- TGGCTAGTTCAGCAGGAA
miR221	F- AGCTACATTGTCTGCTGGGTTTC
miR296	F-GGGTTGGGTGGAGGCTCT
miR320c	F- AAAAGCTGGGTTGGAGGGT

Supplementary Table S2. Evaluation by qRT-PCR of a panel of uEV miRNAs linked to in renal cell damage in the urine of DN patients divided in normoalbuminuric (NAlb; n=8), microalbuminuric (MiAlb; n=5), and macroalbuminuric (MaAlb; n=11) and expressed as Relative Quantification (RQ). RNU6B was used as normalizer. ns= non-significant in respect to NAlb.

Patient Group	miR21 (RQ)	miR24 (RQ)	miR221 (RQ)	miR296 (RQ)	miR320c (RQ)
NAlb	1±0.17	1±0.23	1±0.24	1±0.55	1±1.09
MiAlb	0.80±0.23 (ns)	3.11±8.24 (ns)	2.14±4.41 (ns)	1.20±0.00 (ns)	0.81±0.62 (ns)
MaAlb	0.40±0.19 (ns)	1.15±0.58 (ns)	1.15±0.99 (ns)	0.92±4.64 (ns)	1.94±2.68 (ns)

Supplementary Figure S1. (**A)** Relative quantification of miR145 and miR126 in podocytes (PODO-Cells) treated with TGF-β for 24 hours in the millifluidic coculture dynamic system. Untreated podocytes were used as control (CTL). n=3 independent experiments) (* p<0.05 vs CTL). **(B).** Measure of miR126 and miR145 levels in glomerular endothelial cells (GEC-Cells) untreated (CTL) or treated with TGF-β for 24 hours. (n=3 independent experiments).

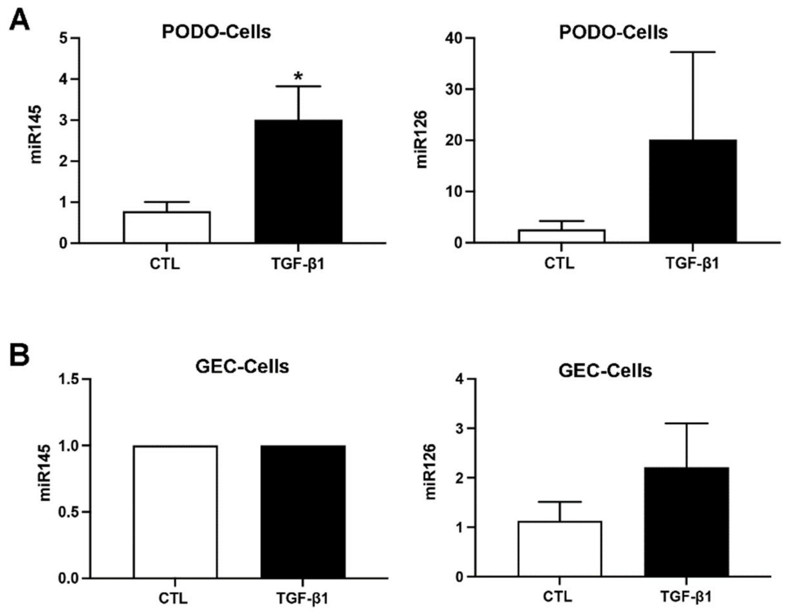

Supplement: Supplementary file 1 [file ijms-23-12098-s001.zip › ijms-1886794-supplementary.pdf]
